# Strain Dependence of Energetics and Kinetics of Vacancy in Tungsten

**DOI:** 10.3390/ma13153375

**Published:** 2020-07-30

**Authors:** Zhong-Zhu Li, Yu-Hao Li, Qing-Yuan Ren, Fang-Fei Ma, Fang-Ya Yue, Hong-Bo Zhou, Guang-Hong Lu

**Affiliations:** 1 Department of Physics, Beihang University, Beijing 100191, China; lizhongzhu@buaa.edu.cn (Z.-Z.L.); buaalyh@163.com (Y.-H.L.); renqy@buaa.edu.cn (Q.-Y.R.); mafangfei0816@buaa.edu.cn (F.-F.M.); yuefangya@buaa.edu.cn (F.-Y.Y.); lgh@buaa.edu.cn (G.-H.L.); 2 Beijing Key Laboratory of Advanced Nuclear Materials and Physics, Beihang University, Beijing 100191, China

**Keywords:** vacancy, hydrostatic/biaxial strain, energetics and kinetics, tungsten

## Abstract

We investigate the influence of hydrostatic/biaxial strain on the formation, migration, and clustering of vacancy in tungsten (W) using a first-principles method, and show that the vacancy behaviors are strongly dependent on the strain. Both a monovacancy formation energy and a divacancy binding energy decrease with the increasing of compressive hydrostatic/biaxial strain, but increase with the increasing of tensile strain. Specifically, the binding energy of divacancy changes from negative to positive when the hydrostatic (biaxial) tensile strain is larger than 1.5% (2%). These results indicate that the compressive strain will facilitate the formation of monovacancy in W, while the tensile strain will enhance the attraction between vacancies. This can be attributed to the redistribution of electronic states of W atoms surrounding vacancy. Furthermore, although the migration energy of the monovacancy also exhibits a monotonic linear dependence on the hydrostatic strain, it shows a parabola with an opening down under the biaxial strain. Namely, the vacancy mobility will always be promoted by biaxial strain in W, almost independent of the sign of strain. Such unexpected anisotropic strain-enhanced vacancy mobility originates from the Poisson effect. On the basis of the first-principles results, the nucleation of vacancy clusters in strained W is further determined with the object kinetic Monte Carlo simulations. It is found that the formation time of tri-vacancy decrease significantly with the increasing of tensile strain, while the vacancy clusters are not observed in compressively strained W, indicating that the tensile strain can enhance the formation of voids. Our results provide a good reference for understanding the vacancy behaviors in W.

## 1. Introduction

Nuclear fusion energy is a good way to relieve the energy shortage in the future, which is being developed internationally via the International Thermonuclear Experimental Reactor (ITER) Project. The choice of the plasma facing materials (PFMs) is one of the critical issues for the steady operation of the future nuclear fusion device [1,2,3]. Tungsten (W) and W alloys are considered as one of the most promising candidates for PFMs, because of its excellent thermal performances and intrinsic structure characteristics [4,5]. However, serving as the PFMs, W will be exposed to high fluxes H isotopes and helium (He) ions as well as high energy neutron. Defects and impurities introduced by these irradiations will seriously degrade the properties and performances of W, leading to the surface blistering, void formation, and irradiation hardening [6,7,8]. Therefore, the behaviors of defects and impurities in W have been under intensive investigations [9,10,11].

Vacancy is the typical intrinsic and radiation-induced defect in materials. Indeed, the presence of vacancy has extremely detrimental effects on the microstructure and properties of W. On the one hand, vacancies can aggregate to form voids, resulting in the swelling, hardening, and embrittlement [12,13,14]. For instance, a large number of voids was observed in W after neutron irradiation at 0.6 dpa and 997 K, leading to the increase of Vickers hardness from 3.61 GPa to 6.68 GPa [8]. On the other hand, vacancies can serve as the strong trapping centers for impurities in W [15,16,17,18], because of the large available space and low electron density. In our previous study [15], the vacancy trapping mechanism for H bubble formation in W has been proposed, which can also be applied to the collection of H in other metals [19,20,21]. Therefore, the formation, migration, and aggregation of vacancy play a key role in the performance of W-PFM and have attracted much attention. It is found that the formation energy and migration energy of a monovacancy in W are 3.1~3.6 eV [22,23,24,25] and 1.6~1.8 eV [24,26,27], respectively. Interestingly, although the binding energy of vacancy cluster is positive, that of a first nearest neighboring (1NN) divacancy in W is negative (−0.12 eV [28,29]), indicating the repulsive interaction between two monovacancies [24,29,30,31]. Nevertheless, vacancy clusters (or voids) have been clearly observed in experiments [8,14]. To remedy this discrepancy, impurities have been considered as a hinge to drive the initial clustering of vacancies, such as carbon, nitrogen, and oxygen [32,33]. However, the negative binding energy of divacancy in pure W remains poorly understood.

Generally, the formation of vacancy will inevitably induce local distortion. Hence, it is believed that the applied strain can significantly affect the behaviors of vacancy. As demonstrated in previous studies [23,34], vacancy formation energy in W, ZnO, and BN is reduced by compressive strain, indicating the high vacancy concentration in the compression region. Further, the migration behavior of vacancy is also altered dramatically by external strain in UO_2_ and CeO_2_, which can be rationalized by the elastic dipole tensor of the transition state (saddle point) with respect to the ground state [35,36]. As a matter of fact, W-PFMs will be subjected to either macroscopic or microscopic deformation under the operational condition. For example, W undergoes significant thermal expansion at elevated temperatures, that is, 0.26%, 1.41%, and 4.64% at 298, 1205, and 2774 K, respectively [37]. Besides, the large temperature gradient inside the W-PFMs also induces thermal stress [38]. More importantly, both intrinsic defects (dislocations and grain boundaries) and radiation-induced defects (dislocation loops, H/He bubbles, precipitates, and so on) induce the long-range stress field as well [39]. Therefore, it is of great importance to determine the influence of strain on the behaviors of vacancy in W.

In the present work, we investigated the influence of isotropic/anisotropic strain on the formation, migration, and clustering of vacancy in W using the first-principles method. The obtained results were further employed in parameterizing object kinetic Monte Carlo (OKMC) model to simulate the dynamical evolution of vacancies in W. Our calculations suggest that vacancy cluster is energetically favorable to nucleate under tensile strain rather than compressive strain, which provides a good reference for understanding the vacancy behaviors in strained W.

## 2. Computational Methods

Our calculations were performed using the Vienna Ab initio Simulation Package (VASP v5.3.5) code [40,41], which carried out self-consistent density functional theory (DFT) with plane-wave pseudopotential. The projector augment-wave method (PAW) was used to describe the interaction between ions and electrons [42]. For the exchange-correlation part, we used the generalized gradient approximation (GGA) proposed by Perdew and Wang [43]. The cutoff energy was set to be 350 eV. Through the convergence tests, a 4a_0_ × 4a_0_ × 4a_0_ body centered cubic (bcc) supercell was used, and the Brillouin zone was sampled with 3 × 3 × 3 *k*-points by the Monkhorst–Pack scheme [44]. During the calculation, both the atomic positions and supercell size/shape are fully relaxed, unless otherwise stated. The calculated equilibrium lattice constant for bcc W is 3.175 Å, in agreement with the previous study [24]. Further, under hydrostatic strain, the lattice parameters of supercell were fixed at given strain values, that is, $\varepsilon_{\mathrm{xx}}=\varepsilon_{yy}=\varepsilon_{zz}=\varepsilon_{set}$. As for anisotropic biaxial strain, the in-plane x and y lattice parameters were fixed at given strain values, that is, $\varepsilon_{\mathrm{xx}}=\varepsilon_{yy}=\varepsilon_{set}$, while the z lattice parameter was relaxed. The energy minimization was continued until the forces on all atoms were less than 0.01 eV/Å. The vacancy migration behavior was determined using a drag method [45,46].

The object kinetic Monte Carlo (OKMC) method is efficient to simulate the defects’ evolution in materials [47,48,49]. In the present work, OKMC is employed to explore the evolution of vacancies in W under different strain conditions, which is parameterized by our DFT results. The fundamental hypotheses and physical models of our OKMC code are the same as those described in [47] and references therein. A cubic box with side length of 157 a_0_ (a_0_ is the lattice constant) is adopted, with each axis parallel to a < 100 > direction of the crystal. Periodic boundary conditions (PBCs) are applied in all directions to mimic an infinite bulk bcc material. Twenty independent simulations were carried out for each case, and the average results are presented in the following parts. Here, in order to compare with the experimental results [50], the temperature was set to be 573 K.

## 3. Results and Discussion

### 3.1. Effects of Strain on the Vacancy Formation

**Figure 1:**
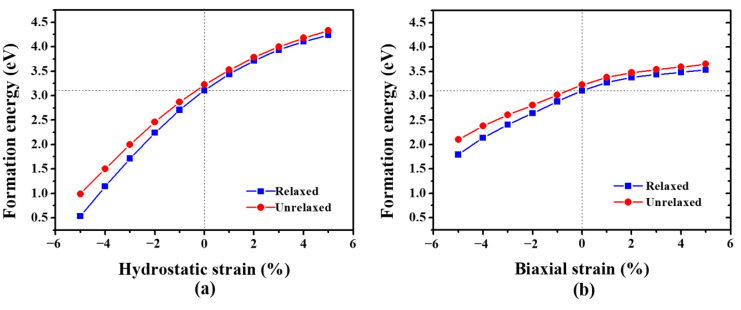
The formation energy of a monovacancy in W under (**a**) hydrostatic strain and (**b**) biaxial strain. The atoms are either fully relaxed (blue square) or fixed (red circle) in the calculations.

In order to investigate the effects of strain on the vacancy formation in W, we calculate the formation energy of a monovacancy under different strain conditions. The formation energy of the monovacancy under strain ε can be expressed as

(1) $$E_{V,\varepsilon }^{f}=E_{N-1,1;\varepsilon }-\frac{N-1}{N}E_{N,0;\varepsilon },$$

where $E_{N,N_{V};\varepsilon }$ is the total energy of the strained system containing *N* W atoms and $N_{V}$ vacancies. According to our calculations, the vacancy formation energy in strain-free W is 3.11 eV, consistent with the previous studies [24,51,52]. Figure 1 shows the formation energy of a monovacancy in strained W. Under both hydrostatic and biaxial strain, the formation energy of a monovacancy increases with the increasing of tensile strain, while it decreases with the increasing of compressive strain. This suggests that the compressive (tensile) strain facilitates (suppresses) the formation of vacancy in W. Furthermore, as displayed in Figure 1, the variation of formation energy under hydrostatic strain is much higher than that under biaxial strain.

Generally, the formation energy of a monovacancy can be divided into two parts, as the vacancy formation is accompanied by the breaking of metallic bonds and the distortion of the lattice [53,54]. The first part is the electronic contribution including both the breaking of metallic bonds and subsequent electronic relaxation. The other is the mechanical contribution induced by the atomic relaxation. Here, in order to eliminate the mechanical contribution, the vacancy formation energy without atomic relaxation (unrelaxed case) is also examined. As shown in Figure 1, the vacancy formation energy in the unrelaxed case is slightly higher (< 0.3 eV) than that in the relaxed case, indicating that the mechanical contribution has little effect on the formation energy of a monovacancy in W. This is because of the small atomic displacement induced by the vacancy formation. For example, the 1NN W atoms shift towards the vacant site by less than 1% in strain-free W. The small atomic displacement can be interpreted by the strong and directional covalent bonds between W atoms, which maintain the rigid-like lattice [55]. Therefore, the electronic contribution plays a dominating role in the formation energy of a monovacancy in W.

**Figure 2:**
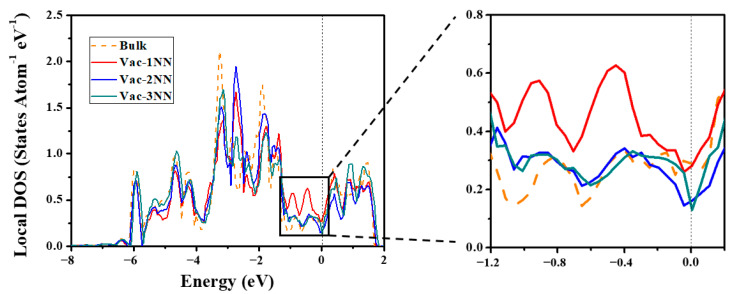
The local density of states (LDOSs) projected on the 5*d* orbitals of a bulk W atom (orange dashed line) and 1NN (red solid line), 2NN (blue solid line), and 3NN (green solid line) W atom of the monovacancy in strain-free case. The Fermi level is denoted by the black dashed line.

Physically, the electronic contribution is closely related to the variation of electronic states. Hence, we further plot the local density of states (LDOSs) projected on the 5*d* orbitals of a bulk W atom and the 1NN, 2NN, and 3NN W atoms surrounding the monovacancy in the strain-free case, as displayed in Figure 2. For the bulk W atom, the Fermi level is close to the minimum of the pseudogap, which well separates bonding states from anti-bonding states. However, the LDOSs of 1NN W atom in the vicinity of the monovacancy exhibit two new peaks in the pseudogap. This indicates that the vacancy formation will affect the electronic states of 1NN W atoms. As shown in Figure 2, the number of electronic states of 1NN W close to the Fermi level are increased significantly, leading to an increase in the total energy of the system. This should be responsible for the relatively high formation energy of a monovacancy in W. Moreover, as illustrated in Figure 2, the LDOSs of 2NN and 3NN W are well consistent with that of a bulk W in the psuedogap, implying that the vacancy-induced variation of electronic states is limited to the 1NN W. Therefore, only the electronic states of 1NN W atoms are examined for the strained cases.

**Figure 3:**
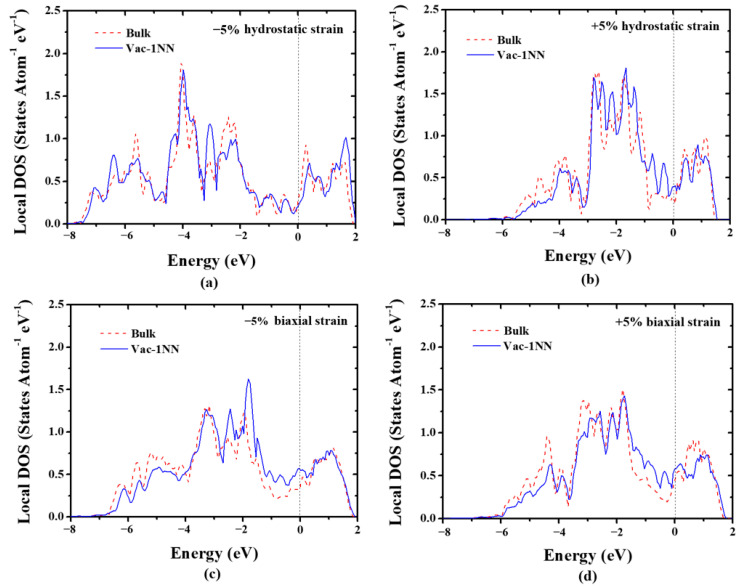
The LDOSs projected on the 5*d* orbitals of a bulk W atom and the 1NN W atom surrounding the monovacancy under different strain conditions. (**a**) 5% compressive hydrostatic strain, (**b**) 5% tensile hydrostatic strain, (**c**) 5% compressive biaxial strain, and (**d**) 5% tensile biaxial strain. The Fermi level is denoted by the black dashed line.

Figure 3 shows the LDOSs projected on the 5*d* orbitals of a bulk W atom and the 1NN W atom surrounding the monovacancy in strained W. Under hydrostatic strain (Figure 3a,b), the energy width of the 5*d* electrons increases under compressive strain, but decreases under tensile strain, which is consistent with the previous study [56]. Interestingly, the vacancy-induced peaks at the pseudogap near the Fermi level for 1NN W atom almost disappear under compressive strain (−5%) (Figure 3a). Therefore, the electronic states’ distribution near the pseudogap of 1NN W atom is almost the same as that of W atom in the bulk system, leading to the significant reduction of the formation energy of a monovacancy. On the contrary, Figure 3b shows that the strain-induced peaks at the pseudogap for the 1NN W become more significant under 5% tensile strain, in comparison with that of the strain-free case. Hence, the formation energy of a monovacancy is increased under tensile strain. Similar results are also observed under biaxial strain, as demonstrated in Figure 3c,d. Moreover, the LDOSs variation of 1NN W atom induced by hydrostatic strain is much larger than that induced by biaxial strain, which is also consistent with the strain-induced variation of vacancy formation energy (see Figure 1). Consequently, these results suggest that the applied strain can significantly affect the electronic states of W atoms nearby, and thus the formation energy of a monovacancy in W.

### 3.2. Effects of Strain on the Vacancy Migration

**Figure 4:**
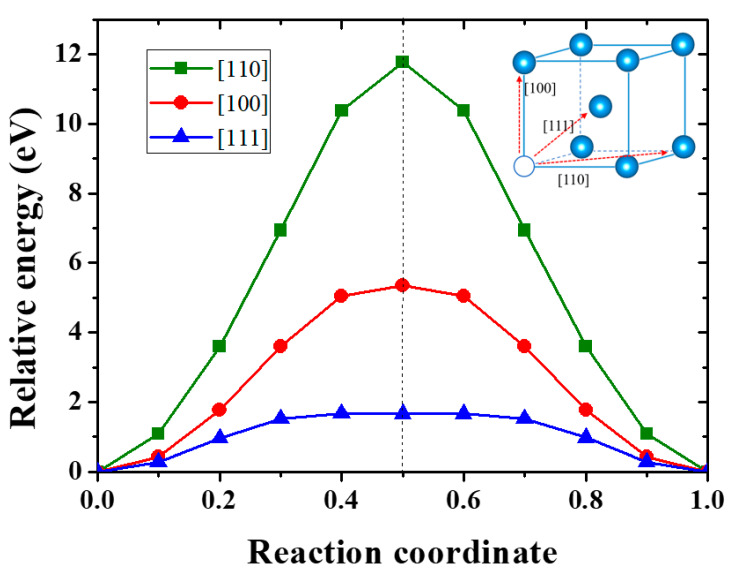
Energy profile for vacancy migration along the [111], [100], and [110] directions in unstrained W.

The mobility of vacancy in materials plays a crucial role on the vacancy evolution and is mainly controlled by the migration energy, which is the energy difference between the transition and ground states. There are three potential migrating directions for a monovacancy in bcc W, that is, < 111 >, < 100 >, and < 110 > directions, and the saddle point lies in the halfway of the migration path for all cases. As shown in Figure 4, the migration energies of a monovacancy in strain-free W along [111], [100] and [110] directions are 1.64 eV, 5.34 eV, and 11.77 eV, respectively. This suggests that the migration along < 111 > direction is the optimal migration path for a monovacancy in W, in agreement with previous results [23,24,25]. As the different paths have the same ground state, the difference of their energy barriers originates from the different structure of transition states. It is found that the interatomic distances between the migratory-W (Mig-W) atom and its 1NN W atoms in transition states are 2.68 Å, 2.47 Å, and 2.22 Å along < 111 >, < 100 >, and < 110 > directions, respectively. These values are much shorter than the equilibrium distance between W atoms (2.75 Å) in perfect lattice. Therefore, the smaller the W-W distance, the larger the lattice distortion in transition states and the higher the migration energy.

**Figure 5:**
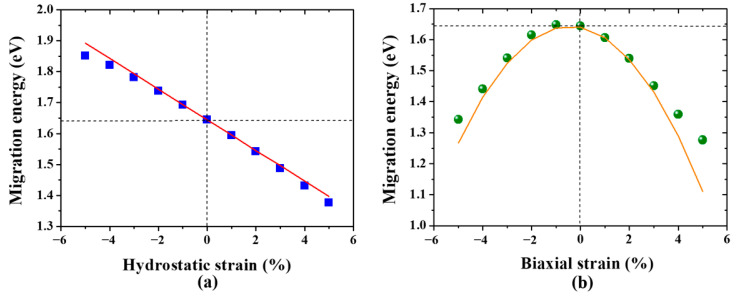
The migration energy of a monovacancy in W under (**a**) hydrostatic strain and (**b**) biaxial strain. The predicted results (red and orange solid line) from elastic dipole tensor are also presented.

Next, we investigate the effect of hydrostatic strain on the migration energy of a monovacancy in W. Owing to the low energy barrier, only the migration of a monovacancy along < 111 > direction was considered. Under hydrostatic strain, the vacancy migration energy decreases “monotonically” with the increasing of tensile strain and increases with the increasing of compressive strain, as shown in Figure 5a. This is in a good agreement with the previous study [23]. Hence, the mobility of a monovacancy in W is promoted by the hydrostatic tensile strain, while it is hindered by the hydrostatic compressive strain.

**Figure 6:**
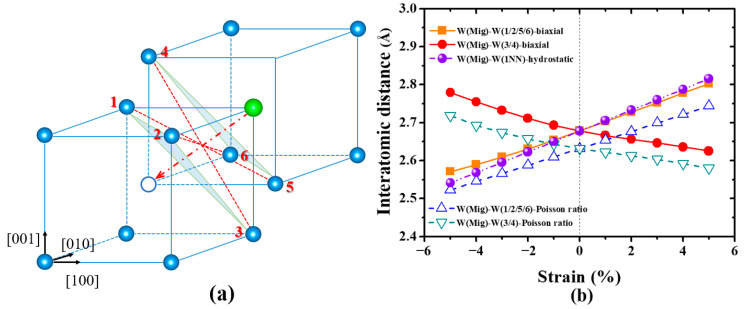
(**a**) The atomic structure during the migration of a monovacancy in W. The green sphere and white circle represent the Mig-W atom and vacant site, respectively. (**b**) The interatomic distances between the Mig-W and its NN atoms for the transition state. The dash dot/solid line corresponds to hydrostatic/biaxial strain, respectively. The dashed line is deduced according to the Poisson ratio.

As the migration energy of a monovacancy in W is strongly related to the atomic structure of transition state (Figure 6a), the interatomic distances between Mig-W and its 1NN W atom under different strain are examined. As illustrated in Figure 6b, the distance of Mig-W-W(1NN) also displays a linear monotonic dependence on hydrostatic strain, that is, the larger the tensile strain, the longer the Mig-W-W(1NN) distance. Thus, the tensile strain facilitates the Mig-W atom passing through the saddle point, resulting in the reduction of migration energy of a monovacancy in W (Figure 5a). In contrast, the Mig-W-W(1NN) distance is shortened under the compressive strain, leading to the increase of migration energy.

In addition, the strain-induced variation of migration energy for a monovacancy can also be rationalized by the elastic dipole tensor. According to the elastic theory, the elastic field induced by the defect will interact with external strain field, thus affecting the formation energy of the defect. For a system subjected to an arbitrary strain state ε, the variation of defect formation energy can be expressed as [35]

(2) $$\Delta E=-tr(G\cdot \varepsilon )=-G_{ij}\varepsilon_{ij},$$

where $G_{ij}$ is the second-rank elastic dipole tensor induced by the defect and $\varepsilon_{ij}$ is the external strain tensor. Both of them are symmetric tensors and the right-hand side of Equation (2) is expressed in Einstein summation convention. The elastic dipole tensor $G_{ij}$ can be obtained through DFT calculation via the strain-controlled approach [35]. In this approach, the defect is introduced in the supercell without the relaxation of the supercell size and shape. After that, the supercell with a defect contains a finite stress $\sigma_{ij}$, and the elastic dipole tensor $G_{ij}$ is given by

(3) $$G_{ij}=V_{0}\sigma_{ij},$$

where V_0_ is the equilibrium volume of the supercell. Thus, the migration energy under given strain state ε can be estimated as

(4) $$\begin{array}{ll} E_{strained}^{mig} & =E_{strained}^{transition}-E_{strained}^{ground}\\ & =(E_{unstrained}^{transition}-G_{ij}^{transition}\varepsilon_{ij}^{})-(E_{unstrained}^{ground}-G_{ij}^{ground}\varepsilon_{ij}^{}) \end{array}$$

where $E_{strained}^{transition}$/$E_{strained}^{ground}$ and $E_{unstrained}^{transition}$/$E_{unstrained}^{ground}$ are energies of the system at the transition/ground state with and without strain, respectively. Note that the shape and volume of the supercell are fixed during the migration of a monovacancy in strained W. Hence, Equation (4) can be further written as

(5) $$E_{strained}^{mig}=E_{unstrained}^{mig}-V_{0}(\sigma_{ij}^{transition}-\sigma_{ij}^{ground})\varepsilon_{ij}^{},$$

where $\sigma_{ij}^{transition}/\sigma_{ij}^{ground}$ is the global stress induced at the transition/ground state, respectively. Therefore, the variation of migration energy under strain depends on the defect-induced stress at the transition state with respect to the ground state.

**Figure 7:**
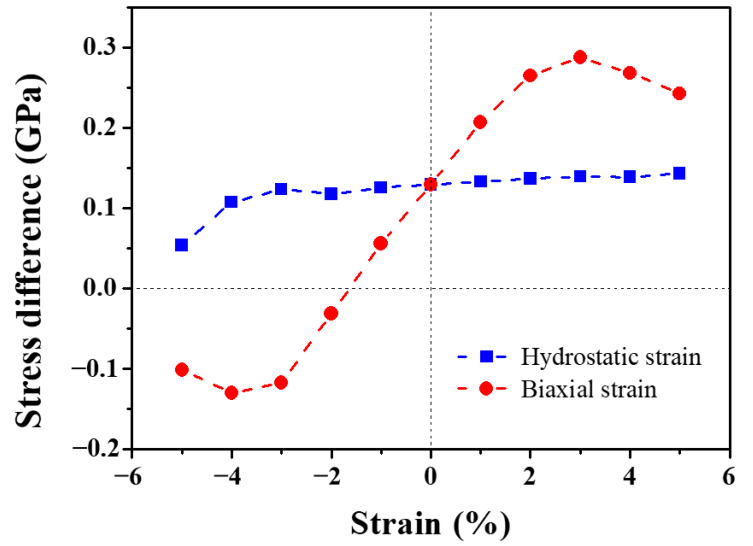
The stress difference between the transition and ground states along the strained direction for hydrostatically (blue dashed line) and biaxially (red dashed line) strained system.

Under hydrostatic strain, three diagonal elements of the elastic dipole tensor for the transition/ground state are the same considering the symmetry of the bcc system, namely $P_{11}^{transition}=P_{22}^{transition}=P_{33}^{transition},\;P_{11}^{ground}=P_{22}^{ground}=P_{33}^{ground}$. Therefore, the stress difference between the transition and ground states along the hydrostatically strained direction are the same, that is, $\left(\sigma_{11}^{transition}-\sigma_{11}^{ground})=(\sigma_{22}^{transition}-\sigma_{22}^{ground})=(\sigma_{33}^{transition}-\sigma_{33}^{ground}\right)$. As illustrated in Figure 7, the stress differences during migration barely change with the hydrostatic strain, except at large compressive strain where nonlinear effects become important. Hence, the stress difference could be approximated as a positive constant, leading to a linear function of the migration energy of a monovacancy on the hydrostatic strain. In this case, Equation (5) can be expressed as

(6) $$E_{strained}^{mig}=E_{unstrained}^{mig}-3V_{0}(\sigma_{11}^{transition}-\sigma_{11}^{ground})\varepsilon_{11}.$$

The positive value of $\left(\sigma_{11}^{transition}-\sigma_{11}^{ground}\right)$ suggests the lattice expansion during the Mig-W atom migrating from the ground state to the transition state. Thus, the extension of lattice under tensile strain balances the expanding tendency, decreasing the migration energy of a monovacancy, while the compressive strain leads to the increase of the migration energy. Indeed, as displayed in Figure 5a, there is a good agreement between the elastic dipole tensor prediction by Equation (6) (solid line) and the direct DFT calculation (data points).

Further, it is important to note that the strain field in W-PFMs should be highly nonuniform and anisotropic owing to the low-symmetry structures of defects and the irregular shape of radiation damage. Therefore, the influence of anisotropic strain on vacancy migration should be considered to understand the evolution of vacancies in W-PFMs. However, little work has focused on this. We further examined the vacancy migration behavior in W under biaxial strain. Figure 5b shows the migration energy of a monovacancy in W under biaxial strain. Surprisingly, the function relation between migration energy and biaxial strain is a *downward parabola* rather than a conventional linear function. Namely, the migration energy of a monovacancy always decreases with the increasing of both tensile and compressive strain, except for a negligible increase (< 0.01 eV) in a limited range of compressive strain. These results suggest that the vacancy migration is effectively promoted by the biaxial strain, independent of its sign.

In order to explore the physical origin for this unexpected result, the atomic configurations for vacancy migration under biaxial strain at transition state were examined. As mentioned above, the Mig-W has six 1NN W atoms with the same equilibrium distance at transition state in the strain-free W (Figure 6a). This symmetric structure remains unchanged under the isotropic strain, and then the distances between Mig-W and its 1NN W are always the same and show a linear relationship with hydrostatic strain (Figure 6b). However, the lattice symmetry is broken by the biaxial strain owing to the Poisson effect. Hence, despite the linear dependence of a specific Mig-W-W distance on the biaxial strain, two opposite slopes of those functions are obtained, as illustrated in Figure 6b. Although the distances between Mig-W and four 1NN W (1/2/5/6 in Figure 6a) atoms show the same trend as that under hydrostatic strain, that is, the distance of Mig-W-W (1/2/5/6) increases (decreases) with the increasing of tensile (compressive) strain, the remaining Mig-W-W (3/4) distances show a completely opposite trend (Figure 6a). This indicates that the lattice distortion is always partly released along the stretched directions under both compressive and tensile strains, leading to the reduction of vacancy migration energy. To quantitatively describe this effect, we employed a simplified statistical approach to estimate the variation of interatomic distances under biaxial strain. As mentioned in Part 2, the in-plane x and y lattice parameters were fixed at given strain values under biaxial strain, that is, $\varepsilon_{xx}=\varepsilon_{yy}=\varepsilon_{set}$, while the z lattice parameter was fully relaxed to $\varepsilon_{zz}$. Thus, the Poisson ratio in the biaxial case can be defined as

(7) $$\upsilon =-\frac{\varepsilon_{zz}}{\varepsilon_{\mathrm{xx}}},$$

which can be obtained by DFT calculations. On the basis of this ratio, the interatomic distance between Mig-W and W-1/2/5/6 is given by

(8) $$d_{1}=\sqrt{{\left[\frac{3}{4}a_{0}(1+\varepsilon_{xx})\right]}^{2}+{\left[\frac{1}{4}a_{0}(1+\varepsilon_{xx})\right]}^{2}+{\left[\frac{1}{4}a_{0}(1-\upsilon \varepsilon_{xx})\right]}^{2}}.$$

Analogously, the interatomic distance between Mig-W and W-3/4 can be expressed as

(9) $$d_{2}=\sqrt{{\left[\frac{1}{4}a_{0}(1+\varepsilon_{xx})\right]}^{2}+{\left[\frac{1}{4}a_{0}(1+\varepsilon_{xx})\right]}^{2}+{\left[\frac{3}{4}a_{0}(1-\upsilon \varepsilon_{xx})\right]}^{2}}.$$

As shown in Figure 6b, the slope of the curves predicted by the statistical approach is well consistent with that obtained by DFT calculations, confirming the critical role of the Poisson effect. Besides, it should be noted that the interatomic distance of the Mig-W-W pair in DFT calculations is slightly higher than that in our simplified statistical approach. This can be attributed to the strong repulsive interaction between Mig-W and its 1NN W atoms at the saddle point of vacancy migration, which leads to the increase of Mig-W-W distance (as the interatomic positions are relaxed spontaneously during DFT calculations) and is not considered in the statistical approach.

The abnormal migration energy of a monovacancy under biaxial strain can also be understood by the stress difference between the transition and ground states. As shown in Figure 7, instead of being invariant under hydrostatic strain, the stress difference increases linearly under small biaxial strain (−3% < ε < +3%). Beyond this region, the nonlinear effects become important. Hence, by analogy with Equation (5), the migration energy under biaxial strain can be obtained by

(10) $$E_{strained}^{mig}=E_{unstrained}^{mig}-V_{0}\int (\sigma_{ij}^{transition}-\sigma_{ij}^{ground})d\varepsilon_{ij}^{}.$$

Obviously, the integration of a liner function results in a quadratic function, corresponding to the parabolic dependence of vacancy migration energy on biaxial strain. Indeed, as displayed in Figure 5b, there is a good agreement between the elastic dipole tensor prediction by Equation (7) (solid line) and the DFT calculation (data points).

### 3.3. Effects of Strain on the Interaction between Two Vacancies

The interaction between vacancies plays a key role in the nucleation of vacancies in materials. In order to explore the influence of strain on the nucleation of vacancies, we investigate the interaction between two vacancies (divacancy) under hydrostatic and biaxial strain in W. The binding energy of divacancy under strain ε is defined as the energy released during the coalescence of two monovacancies, which can be expressed as

(11) $$E_{\varepsilon }^{b}=2E_{N-1,1;\varepsilon }-E_{N-2,2;\varepsilon }-E_{N,0;\varepsilon }.$$

The positive values of the binding energy indicate attractive interaction, while the negative ones refer to repulsive interaction.

In strain-free W, the binding energies of divacancy along the [111] (1NN) and [100] (2NN) directions are −0.09 eV and −0.43 eV, respectively. This indicates that two vacancies with 1NN distance exhibit weak repulsion, and the repulsive interaction becomes stronger at 2NN distance. These results are consistent with previous studies [28,29]. To understand the contribution of electronic and mechanical relaxation to the interaction of divacancy, we also calculate the binding energy of 1NN and 2NN divacancy with a fixed atomic structure (unrelaxed case). In this case, the binding energies of 1NN and 2NN divacancy are calculated to be −0.1 eV and −0.43 eV, respectively, which is almost the same as the fully relaxed case. This indicates that the atomic relaxation has little effect on the interaction of divacancy, and thus the electronic contribution plays a dominating role.

**Figure 8:**
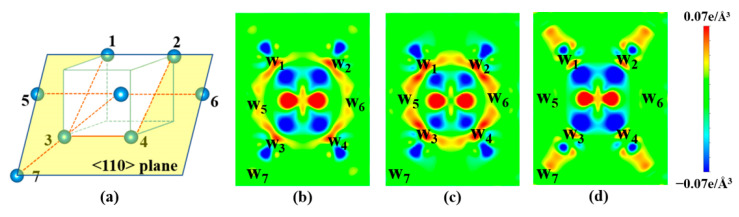
(**a**) The close-packed < 110 > plane across the monovacancy. The differential charge density distributions (in e/Å^3^) of the < 110 > plane under (**b**) zero strain, (**c**) 5% hydrostatic compressive strain, and (**d**) 5% hydrostatic tensile strain.

**Figure 9:**
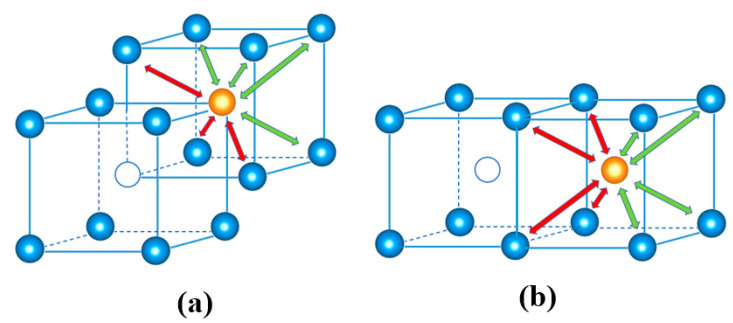
Schematic diagram of bonds breaking when forming the (**a**) 1NN and (**b**) 2NN divacancy. The white circle, blue sphere, and orange sphere denote the pre-existing monovacancy, bulk W atom, and second W atom to be removed, respectively. The green double-sided arrow represents the normal W-W bond, while the red one refers to the enhanced 1NN–2NN bond.

Figure 8 shows the differential charge density on the < 110 > plane, which is the close-packed plane in bcc W. The differential charge density is defined as the difference between the superposition densities of the monovacancy system *plus* a single W atom at the vacant site and the charge density of bulk W. The red region indicates accumulation of electrons after forming a monovacancy, while the blue region refers to depletion of electrons. Figure 8b shows that there is obvious electron accumulation (like a bond ring) between 1NN (e.g., W1) and 2NN (e.g., W5) W atoms. The accumulated electrons form a bonding cage surrounding the monovacancy, which causes more energy to be consumed to further break the metallic bonds in comparison with the perfect W. A similar phenomenon has also been reported in fcc Al [54]. Here, we refer to this enhanced bonding between 1NN–2NN W atoms as 1NN–2NN bonds. For the 1NN divacancy, seven extra bonds are broken for the formation of the second vacancy, as shown in Figure 9a, which is lower than that of a monovacancy (~8 extra bonds). However, three of them are the enhanced 1NN–2NN bonds, denoted as red double-sided arrow (Figure 9a). Moreover, it needs more energy to break these enhanced bonds than to break the four normal bonds. Accordingly, there is slight repulsion for the 1NN divacancy. As for the 2NN divacancy, eight bonds are broken for the formation of the second vacancy and four of them are enhanced 1NN–2NN bonds, as illustrated in Figure 9b. Thus, extra energy is needed to create the second vacancy, leading to the low binding energy of 2NN divacancy. Therefore, we mainly focus on the binding energy of the 1NN divacancy for the strained case, because it is more stable than the 2NN divacancy.

**Figure 10:**
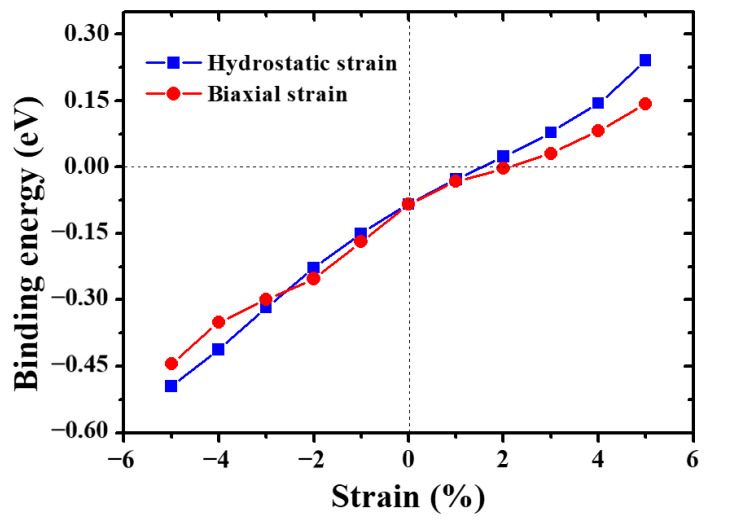
Effects of hydrostatic and biaxial strain on the binding energy of divacancy in W.

Figure 10 shows the variation of binding energy of 1NN divacancy under both hydrostatic and biaxial strain. It can be found that the binding energy of 1NN divacancy increases with the increasing of tensile strain, while it decreases with the increasing of compressive strain. This suggests that tensile strain facilitates the clustering of vacancies, but compressive strain suppresses it. Interestingly, the binding energy turns from negative to positive when the hydrostatic/biaxial tensile strain exceeds 1.5%/2%, respectively. This suggests that the 1NN divacancy is energetically stable under large hydrostatic/biaxial tensile strain.

As mentioned above, the binding energy of divacancy is closely related to the electron redistribution between the 1NN and 2NN W atoms surrounding the monovacancy. Therefore, we further plot the differential charge density on the < 110 > plane under ± 5% hydrostatic strains. Under 5% compressive strain (Figure 8c), the 1NN-2NN bonds become even stronger owing to the more compact atomic configuration. The enhanced bonding cage surrounding the monovacancy makes it harder to remove the second W atom, leading to the decrease of the binding energy. In contrast, under 5% hydrostatic tensile strain, the 1NN-2NN bonds almost vanish (Figure 8d). The weakened bonding cage makes it easier to remove the second W atom, resulting in the increase of the binding energy. Consequently, the strain will affect the vacancy clustering in W by changing the electron distribution surrounding the vacancy.

### 3.4. Effects of Strain on the Nucleation of Vacancies

Recently, Zibrov et al. investigated the effects of plastic deformation on the performance of W exposed to D plasma [50]. It is found that the D retention is enhanced in the deformed W, which can be attributed to the formation of vacancy-type defects induced by deformation. This suggests that the strain has a significant effect on the evolution of vacancies in W. Hence, on the basis of the energetic and kinetics parameters of vacancies under strain, as mentioned above, we further employ the OKMC method to investigate the effect of both hydrostatic and biaxial strain on the nucleation of vacancies in W.

Here, the initial concentration of vacancy is set to be 10^−5^ appm (77 monovacancies), and the monovacancies are randomly inserted in the simulation box. The simulation temperature is 573 K, which is consistent with the experiment [50]. The vacancy object is allowed to hop to one of the eight bcc nearest neighboring lattice sites with the vibration frequency of 6 × 10^12^/s. As the migration energy of the monovacancy and the 1NN divacancy in strain free W is almost the same [25], the migration energy of < 111 > divacancy in strained W is assumed to be the same as that of the monovacancy. As for the tri-vacancy in W, it can migrate rapidly throughout the matrix, with the migration energy of ~0.90 eV [57]. Once a tri-vacancy interacts with another monovacancy, a stable vacancy cluster can be formed with high binding energy (> 0.6 eV in [24]), which is hard to dissociate at 573 K and serves as the trapping center for the subsequent vacancies. Therefore, the formation of tri-vacancy can be used as the sign of vacancy nucleation.

**Figure 11:**
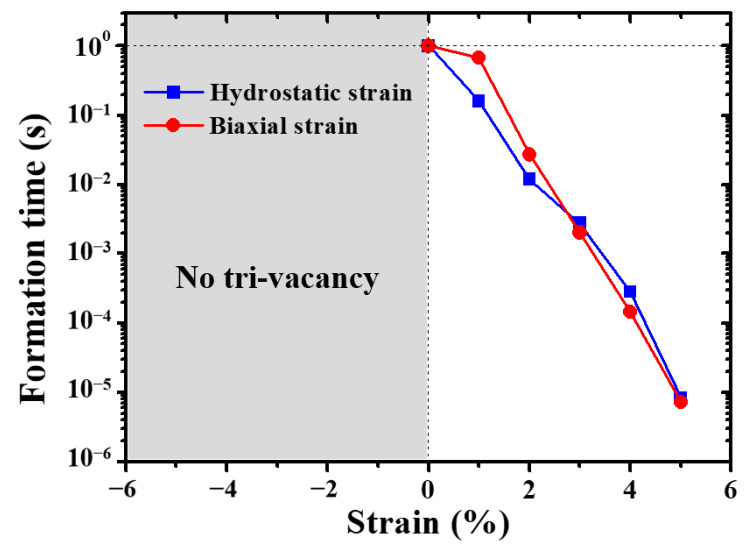
The formation time of tri-vacancy under both hydrostatic and biaxial strains in W.

Figure 11 shows the formation time of tri-vacancy in W under different strain conditions. It is found that the tri-vacancy is observed under both hydrostatic/biaxial tensile strain. The formation time of the tri-vacancy significantly decreases with the increase of tensile strain. However, there is no tri-vacancy under hydrostatic/biaxial compressive strain. This can be attributed to the influence of strain on the mobility and clustering of vacancies in W. For the tensile strain cases, the migration energy of the monovacancy decreases with the increase of strain, enhancing the clustering of vacancies kinetically. Moreover, the binding energy of divacancy is positive under hydrostatic/biaxial tensile strains, except for strain of 1%. This suggests that, once a divacancy is formed, it is unlikely to dissociate. These two factors shorten the formation time of tri-vacancy with the increase of tensile strain, as illustrated in Figure 11. On the contrary, the mobility of the monovacancy is significantly reduced owing to the high migration energy under hydrostatic compressive strain. Therefore, the formation of the divacancy and tri-vacancy requires much more time. In addition, the binding energy of the divacancy is negative under hydrostatic compressive strain, indicating that the divacancy is energetically favorable to dissociate, thus inhibiting the formation of the tri-vacancy. Under biaxial compressive strain, the migration energy of the monovacancy is significantly reduced, corresponding to the high mobility of the monovacancy. However, the binding energy of the divacancy is negative, which indicates that, even though a divacancy is formed, it will soon dissociate before meeting another monovacancy. Therefore, the formation of the tri-vacancy is not observed in the whole simulation time scale under biaxial compressive strains. Consequently, the tensile strain will facilitate the nucleation of vacancies, while the compressive strain will suppress it. Note that, although tri-vacancy is not observed under ε≤0% in our OKMC simulation time scale owing to the negative binding energy, vacancy clusters could indeed be formed owing to the stabilizing effect of impurities (such as carbon and oxygen [32,33]), which is beyond the scope of this work.

## 4. Summary

In summary, we have investigated the formation, migration, and clustering of vacancy in tungsten (W) under different strain conditions using a first-principles method combined with linear elastic theory. The formation energy of a monovacancy responds to hydrostatic strain “monotonically”, that is, increasing (decreasing) with the increase of tensile (compressive) strain. This can be rationalized by the variation of electronic states of the 1NN W atoms surrounding the monovacancy. The ideal path for the migration of a monovacancy is < 111 > direction and the corresponding migration energy decreases monotonically with hydrostatic strain. Surprisingly, the vacancy mobility is always promoted by biaxial strain, almost independent of the sign of strain, which originates from the Poisson effect. Although the binding energy of divacancy is negative in strain-free W, it increases monotonically with both hydrostatic and biaxial strains. Specifically, the binding energy of divacancy turns from negative to positive at ε = 1.5%/2% for hydrostatic/biaxial strain. This indicates that two 1NN monovacancies are energetically favorable to bind with each other under large hydrostatic/biaxial tensile strain, leading to the formation of vacancy clusters. Furthermore, the nucleation of vacancy cluster under different strain conditions is simulated with the OKMC method. The nucleation time of vacancy cluster decreases significantly with the increasing tensile strain, while it is not observed under compressive strain for the whole simulation time. This suggests the enhancing effect of tensile strain on the formation of voids in W, which is consistent with the experimental observations.
